# Understanding the Role of Adipokines in Cardiometabolic Dysfunction: A Review of Current Knowledge

**DOI:** 10.3390/biom15050612

**Published:** 2025-04-23

**Authors:** Sayantap Datta, Saisudha Koka, Krishna M. Boini

**Affiliations:** 1 Department of Pharmacological and Pharmaceutical Sciences, College of Pharmacy, University of Houston, 4349 Martin Luther King Blvd., Houston, TX 77204, USA; sdatta20@cougarnet.uh.edu; 2 Department of Pharmaceutical Sciences, College of Pharmacy, Texas A & M University, Kingsville, TX 78363, USA

**Keywords:** adipokine, inflammation, obesity, atherosclerosis, hypertension

## Abstract

Cardiometabolic risk and associated dysfunctions contribute largely to the recent rise in mortality globally. Advancements in multi-omics in recent years promise a better understanding of potential biomarkers that enable an early diagnosis of cardiometabolic dysfunction. However, the molecular mechanisms driving the onset and progression of cardiometabolic disorders remain poorly understood. Adipokines are adipocyte-specific cytokines that are central to deleterious cardiometabolic alterations. They exhibit both pro-inflammatory and anti-inflammatory effects, complicating their association with cardiometabolic disturbances. Thus, understanding the cardiometabolic association of adipokines from a molecular and signaling perspective assumes great importance. This review presents a comprehensive outline of the most prominent adipokines exhibiting pro-inflammatory and/or anti-inflammatory functions in cardiometabolic dysfunction. The review also presents an insight into the pathophysiological implications of such adipokines in different cardiometabolic dysfunction conditions, the status of adipokine druggability, and future studies that can be undertaken to address the existing scientific gap. A clear understanding of the functional and mechanistic role of adipokines can potentially improve our understanding of cardiovascular disease pathophysiology and enhance our current therapeutic regimen in the years to come.

## 1. Introduction

Metabolic syndrome (MetS) is clinically defined as a cluster of biochemical anomalies that are associated with a broad array of cardiometabolic risk factors such as abdominal obesity, augmented blood pressure, increased triglyceride levels, and/or enhanced fasting blood sugar [1,2]. Studies over the past few years have shown a steep rise in the global prevalence of MetS or cardiometabolic dysfunction [3,4,5]. This has primarily been attributed to a spectrum of metabolic and cardiovascular symptoms such as an increase in childhood obesity and type II diabetes (T2D), hypertension, dyslipidemia, and atherosclerosis [6,7]. In the United States, 29% of youth aged between 2 and 19 years and 19% of the adult population exhibit stable cardiometabolic conditions [8,9]. The subjects at cardiometabolic risk are susceptible to a broad array of suboptimal risk factors over an extended timeline and eventually culminate in reliance on therapy that can control but not reverse the altered cardiometabolic condition [10]. Recent courses of meta-analysis studies largely associate prediabetes (non-diabetic hyperglycemia), non-alcoholic fatty liver disease (NAFLD), and other high-risk metabolic states with an increased risk of MetS occurrence and/or progression [11,12,13]. This presents the dire need to identify novel biomarkers of MetS that ensure the early diagnosis and mitigation of cardiometabolic risk to augment healthy life expectancy beyond current estimates [14]. Despite multi-omics and genomic approaches promising to identify potential biomarkers and a better understanding of MetS occurrence [15], associated molecular mechanisms remain poorly understood.

The adipose tissue is primarily responsible for energy storage and secretes a broad spectrum of regulatory molecules that exhibit autocrine, paracrine, and endocrine functions to maintain metabolic stability [16,17]. Adipokines are immunomodulatory molecules secreted by adipocytes [18]. Studies report that alterations in adipocyte levels are closely associated with chronic inflammatory responses, culminating in cardiometabolic dysfunction [19]. The secretory profile of adipokines is altered in metabolic dysfunction, with an overall increase in the production of pro-inflammatory adipokines such as tumor necrosis factor (TNF)-α, leptin, resistin, angiopoietin-related protein 2 (ANGPTL2), and interleukins (ILs) [20]. This stimulates obesity-associated stress via the activation of the autonomic nervous system (ANS) and the hypothalamus–pituitary–adrenal (HPA) axis [21]. Prolonged ANS and HPA axis stimulation induces a rise in glucocorticoid levels that, in turn, increase white adipose tissue (WAT) mass [22]. The secretion of pro-inflammatory cytokines from increased WAT mass induces pre-adipocyte differentiation and further contributes to maintaining the activation status of the HPA axis [22]. This promotes a deleterious cascade of metabolic and immune responses that induce chronic inflammatory condition and ensure the onset of cardiometabolic dysfunction [22].

Although the majority of the adipokines are pro-inflammatory, studies also identify anti-inflammatory adipokines, chiefly adiponectin, and their beneficial impact against complications associated with MetS [23]. The dysregulation of anti-inflammatory adipokines strongly contributes to local or systemic inflammatory induction and MetS onset [24]. Clinical studies strongly suggest a negative correlation between adiponectin profile and pro-inflammatory C-reactive protein (CRP) and IL-6 levels [25,26]. Adiponectin reduces TNF-α expression through NF-ƘB signaling downregulation [27], attenuates class A scavenger receptor (SR)-A, prevents macrophage foam cell formation [28], and decreases CD4^+^ T lymphocyte infiltration into atherosclerotic lesions via the regulation of macrophage-derived T lymphocyte chemoattractants [29]. However, a clear mechanistic understanding of the role of anti-inflammatory adipokines in MetS conditions remains poorly addressed.

This review attempts to focus on the functional and mechanistic role of pro-inflammatory and anti-inflammatory adipokines in cardiometabolic dysfunction from a molecular signaling perspective. The review also highlights the specific influence of adipokines in regulating the onset and progression of some of the prominent cardiometabolic dysfunctional conditions, such as hypertension, T2D, dyslipidemia, and renal damage, among others. Such functional and mechanistic elucidation of the role of adipokines can enable a better understanding of MetS pathophysiology and potentially improve therapeutic strategies in the years to come.

## 2. Adipokines

**Figure 1:**
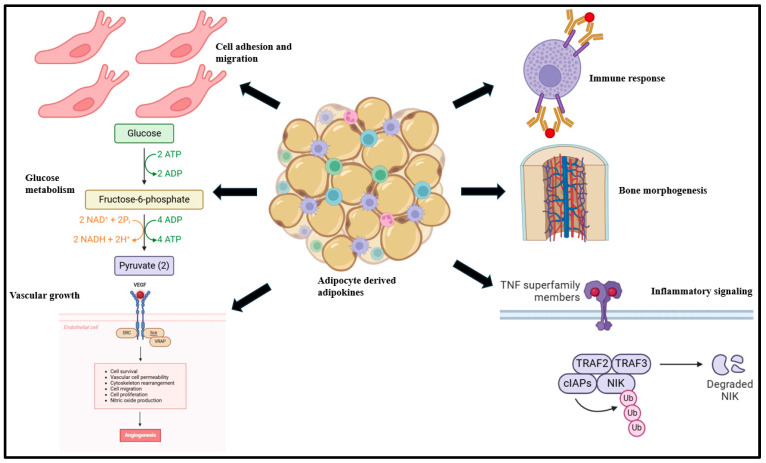
Adipokines, derived primarily from adipocyte tissue, influence a broad variety of biological functions such as immune response, bone morphogenesis, inflammation, cellular adhesion, migration, glucose metabolism, and vascular growth functions [36].

Adipokines are bioactive peptides derived from adipocytes that regulate a broad array of physiological functions, such as body fat distribution, insulin sensitivity, endothelial function, and blood pressure, among others [30,31,32]. In adipose tissue, adipokines contribute to adipogenesis, immune cell migration, and metabolism [33,34,35]. At the systemic level, adipokines influence the biological functioning of the heart, liver, brain, and vasculature [33] and worsen immune response, inflammatory signaling, glucose metabolism, bone morphogenesis, myocardial contractility, and cell adhesion, among others (Figure 1) [35].

### 2.1. Pro-Inflammatory Adipokines

#### 2.1.1. Tumor Necrosis Factor (TNF)-α

**Figure 2:**
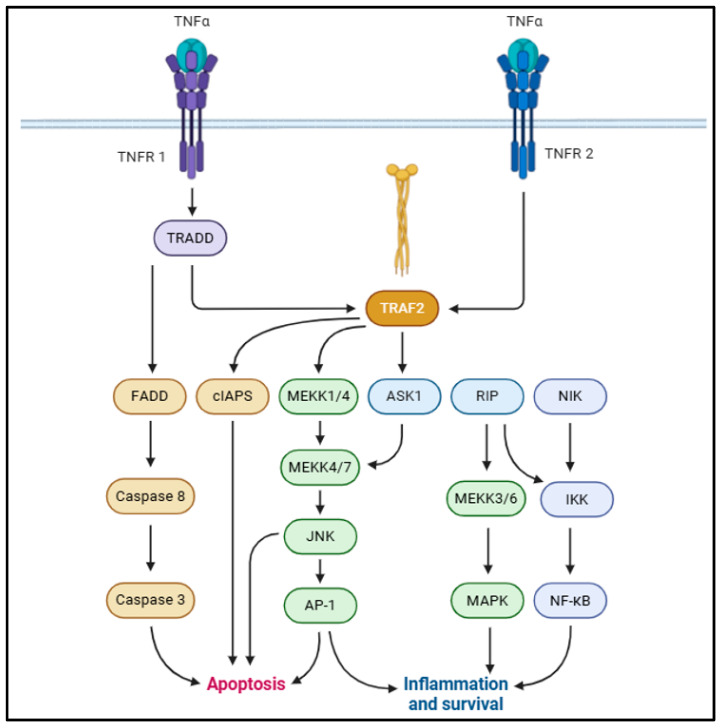
Schematic representation of pro-inflammatory TNF-α signaling, contributing to MetS onset [36].

Tumor necrosis factor (TNF)-α is a 17 kDa proteolytic cleavage product from a 26 kD transmembrane monomer, typically driven by TNF-α-converting enzyme (TACE) [37,38]. TNF-α is associated with a broad array of physiological functions such as immune response, cellular proliferation, differentiation, apoptosis, and energy homeostasis [39,40]. TNF-α is typically derived from macrophages, adipocytes, and visceral fat and bears a positive correlation with hyperinsulinemia, body mass index (BMI), and insulin resistance [41,42]. Studies suggest that TNF-α augments IL-6 production and impairs insulin signaling in adipocytes and hepatocytes [43,44,45]. TNF-α typically correlates with adiposity, increased adipocyte lipolysis, fatty acid release into the bloodstream from adipocytes, and insulin resistance [46,47,48]. This is typically mediated via binding with TNF-α receptor (TNFR)-1 or TNFR-2 and the activation of downstream signaling cascades [49]. The adipogenic effects of TNF-α are majorly mediated via TNFR1 binding, although some studies also suggest regulation via TNFR2 binding [50,51,52]. Upon TNFR1 binding, TNFR-associated death domain protein (TRADD) recruits Fas-associated death domain protein (FADD), TNFR-associated factor 2 (TRAF2), receptor-interacting protein (RIP)-1, and mitogen-activated protein kinase (MAPK), activating death domain protein (MADD) [53,54]. In adipocytes, TNF-α activates extracellular signal-regulated kinase (ERK)-1/2, p38 MAPK, and c-Jun N-terminal kinase (JNK) via MADD [49,55,56]. This drives the phosphorylation of insulin receptor substrate (IRS)-1 and compromises insulin signaling [57,58]. TRAF2 and RIP1 recruit inhibitors of NF-ƘB (IƘB) kinase (IKK) to TNFR1 and activate the NF-ƘB pro-inflammatory signaling pathway (Figure 2) [59,60]. In addition to suppressing IR, IRS-1, and glucose transporter type IV (GLUT4) expression [45,61], TNF-α also suppresses peroxisome proliferator-activated receptor (PPAR)-γ transcription factor in adipocytes [62]. This is mediated primarily via serine phosphorylation of PPAR-γ N-terminal residues, chiefly driven by JNK/ERK1/2 signaling activation [63,64].

#### 2.1.2. Leptin

Leptin is a peptide hormone encoded on chromosome 7 locus that is primarily derived from WAT [65,66]. It is a 16 kDa protein of 167 amino acids that possesses the capabilities to circulate in its free state as well as bound to proteins [67]. It possesses great potential to cross the blood–brain barrier and exercise a significant impact on hypothalamus functioning via the hypothalamic leptin receptor (LEPR) [68,69,70,71]. It regulates satiety, appetite, reproductive functions, fertility, puberty, atherogenesis, and energy expenditure [72]. Leptin plays a significant role in lipogenesis and the expansion of the total fatty acid content of the body and exhibits sex differences in its synthesis potential, where females exhibit a higher rate and extent of leptin synthesis relative to males [48,73,74]. Additionally, leptin reduces insulin secretion and modulates hematopoiesis, β-cell functions of the pancreas, peripheral adiposity, and central nervous system (CNS) functions [75,76,77,78,79]. Cumulatively, these effects are associated with cardiometabolic stability [80,81]. In fact, CNS/leptin signaling disruption has been reported to contribute to cardiometabolic dysfunction such as obesity, hypertension, and T2D [82,83,84]. This is primarily driven by systemic leptin resistance and tolerance in obese patients [85,86].

**Figure 3:**
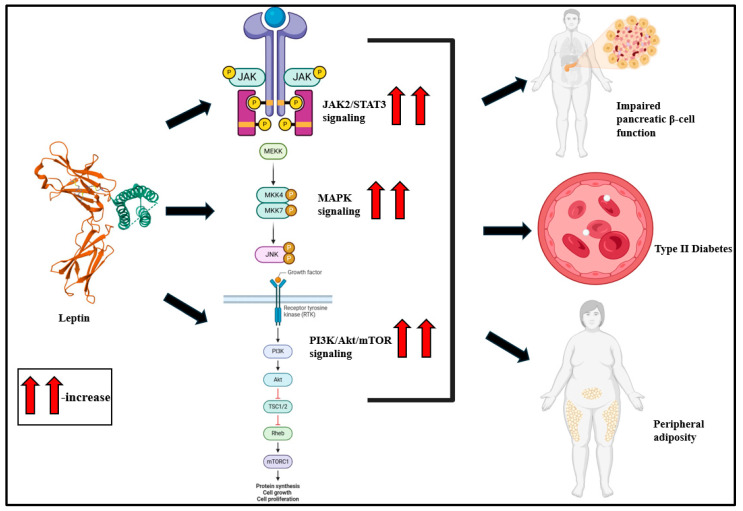
Leptin, a pro-inflammatory cytokine, activates JAK2/STAT3, MAPK, and PI3K/Akt/mTOR signaling and is predisposed to impaired pancreatic β-cell function, T2D, and peripheral adiposity [36,107].

As a pro-inflammatory adipokine, leptin promotes inflammatory cytokine upsurge in macrophages [87,88] and T lymphocytes and triggers inflammatory signaling via the Janus kinase/signal transducer and the activator of transcription-3 (JAK-STAT3) [89,90], MAPK [91,92], and phosphatidylinositol-4,5-bisphosphate3-kinase (PI3K) signaling cascades (Figure 3) [93,94]. The binding of leptin to LEPR activates JAK2, and this phosphorylates Tyr985, Tyr1077, and Tyr1138 in the LEPR cytoplasmic domain [95,96]. Upon phosphorylation, STAT3 translocates from the cytoplasm to the nucleus, binds to the *pomc* promoter, and stimulates proopiomelanocortin (POMC) [90,97]. Leptin-associated PI3K/Akt activation is typically mediated via IRS phosphorylation [98,99]. This stimulates the mammalian target of rapamycin (mTOR), typically via p70S6 kinase phosphorylation [100,101].

Randomized controlled trials (RCTs) in hypothalamic amenorrhea (HA) women (low circulating leptin) suggested controversial perspectives on leptin administration [102]. One RCT suggested that leptin augments osteocalcin and urine-N-terminal telopeptide but had no impact on bone mineral density [103]. However, another RCT suggested that leptin administration improved lumbar spine bone mineral density in hypoleptinemic women [104]. Thirty-six weeks of leptin administration in HA women culminated in parathyroid hormonal and RANKL/OPG ratio downregulation, pointing to reduced osteoclastic activity [105,106]. These studies keep the central and peripheral impact of leptin on bone mineral density highly debatable.

#### 2.1.3. Visfatin

**Figure 4:**
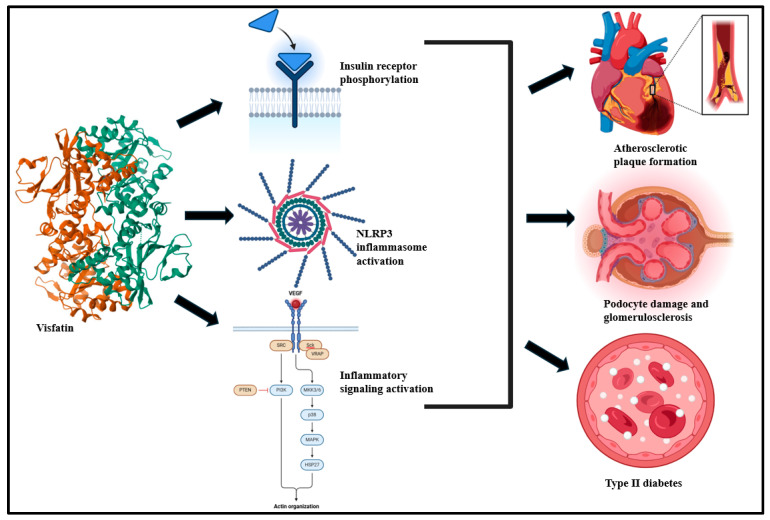
Visceral adipose tissue-derived visfatin drives insulin receptor phosphorylation, NLRP3 inflammasome, and inflammatory signaling activation and culminates in atherosclerosis, podocyte damage, and T2D [36,134].

Visfatin, a 52 kDa pre-B-cell colony-enhancing factor (PBEF) protein, is primarily derived from visceral adipose tissue macrophages [108,109]. Its intracellular form, nicotinamide phosphoribosyl transferase (NAMPT), is a key regulator of nicotinamide adenine dinucleotide (NAD) biosynthesis [110]. Visfatin drives the release of pro-inflammatory mediators and upregulates vascular endothelial growth factor (VEGF), fibroblast growth factor (FGF)-2, matrix metalloproteinase, and monocyte chemoattractant protein-1 (MCP-1) production [111]. This culminates in NF-ƘB, MAPK, and PI3K signaling activation [112]. Studies over the years suggest high plasma circulatory levels of visfatin in patients experiencing MetS, such as obesity and T2D [113,114,115]. High circulatory levels of visfatin have been associated with B-cell deterioration [116,117]. Visfatin plays a role in the pathogenesis of T2D, potentially via interacting with insulin receptors, and it is typically driven by the phosphorylation of insulin substrate-1 and insulin substrate-2 [118]. Studies suggest that visfatin is central to NOD, LRR, and pyrin domain-containing protein 3 (NLRP3) inflammasome activation [119]. The NLRP3 inflammasome activation promotes the extracellular release of damage-associated molecular pattern (DAMP) molecules, like high-mobility group box-1 (HMGB1), and drives podocyte and inter-endothelial injury through the paracrine and autocrine signaling cascade [4,120,121]. This holds the key to arterial inflammation and endothelial damage in a TLR4-dependent manner and culminates in obesity (Figure 4) [122,123].

Clinical studies with 12 healthy men suggested that visfatin levels are elevated by microgravity and remain increased post-recovery [124]. A small cohort study suggested a correlation between visfatin and male lumbar spine bone mineral density [125]. A large group of clinical studies, however, report augmented visfatin tissue expression in sepsis, psoriasis, and other inflammatory states [126,127]. Clinical studies also highlight enhanced serum visfatin in atherosclerosis and coronary artery disease (CAD) patients [128,129]. Clinical studies also show that visfatin levels are raised significantly in gestational T2D patients [130]. This has been confirmed further at different gestational stages, such as the first trimester [131], second trimester [132], and third trimester [133].

#### 2.1.4. Resistin

**Figure 5:**
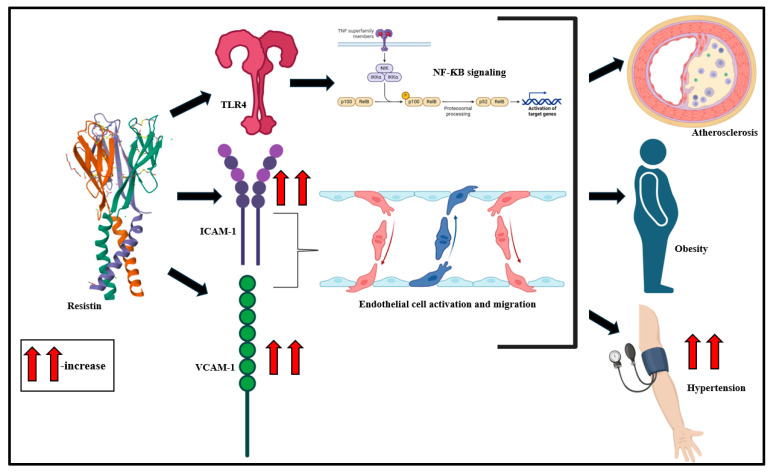
Resistin mediates TLR4-driven NF-ƘB signaling activation, ICAM-1- and VCAM-1-driven endothelial cell stimulation, and migration and culminates in atherosclerosis, obesity, and hypertension [36,154].

Resistin, primarily derived from macrophages, monocytes, and pre-adipocytes, is a polypeptide of 108 amino acids with a molecular weight of 11.3 kDa [135,136,137]. Resistin modulates insulin homeostasis and selectively disables the inhibitory impact of insulin on hepatic glucose generation [138,139,140,141]. Investigations propose that the 420C/G polymorphism of the resistin gene is associated with the high circulatory potential of resistin [142]. Studies suggest that resistin downregulates adenosine monophosphate-activated protein kinase (AMPK) signaling in the liver and skeletal muscle sites, which interferes with insulin signaling [143,144]. Resistin also binds to toll-like receptor (TLR) 4 in the hypothalamus and mediates inflammatory stimulation via the classical NF-ƘB signaling cascade [145,146]. Endothelial cell stimulation is also driven by resistin, typically via the increase in expression of endothelin-1, intercellular adhesion molecule (ICAM)-1, and vascular cell adhesion molecule (VCAM)-1 [147]. Cumulatively, these serve as precursors to atherosclerosis, hypertension, and MetS onset (Figure 5) [148,149,150].

Clinical studies, over the years, highlight a strong association of resistin with insulin resistance in obese individuals [151]. Plasma resistin levels are significantly altered with CVD risk factors and correlate with vascular smooth muscle cellular dysfunction in advanced atherosclerosis and also with intimal hyperplasia [152]. This clinical inflammatory state also involves macrophage infiltration in adipose tissue and the production of VCAM-1, ICAM-1, and MCP-1 [137,153].

#### 2.1.5. Angiopoietin-like Protein (ANGPTL)

Angiopoietin-like protein (ANGPTL) is a family of proteins that exhibit an N-terminal coiled coil domain and a C-terminal fibrinogen-like domain [155,156]. They act differently as compared to structurally similar angiopoietin, as they do not bind to angiopoietin receptors Tie1 or Tie2 [157,158].

##### ANGPTL2

ANGPTL2 is a 57 kDa glycosylated protein member of the ANGPTL family derived from adipose tissue [159,160]. Existing studies suggest that ANGPTL2 expression and circulation are augmented in high-fat diet conditions and associated with local inflammation, an increase in macrophage accumulation, endothelial injury, adipose tissue-specific pro-inflammatory M1 polarization, and systemic insulin resistance [159,161,162]. An ANGPTL2-based inflammatory response is typically guided by the activation of the NF-ƘB signaling pathway [159]. ANGPTL2 binds to α5β1, activates Rac1, degrades IƘB, and promotes the translocation of NF-ƘB to the nucleus [163,164]. Extended NF-ƘB signaling activation by ANGPTL2 affects pancreatic and hepatic functioning and accounts for the development of T2D and insulin resistance [159]. ANGPTL2 also accelerates ICAM-1 and P-selectin expression and mediates atherosclerotic plaque formation [165]. In fact, a rise in circulating ANGPTL2 levels bears a close association with C-reactive protein expression in obesity and an increase in expression of pro-inflammatory TNF-α and its receptor TNFR1 in the onset and progression of T2D, obesity, and heart failure [159,166,167].

##### ANGPTL4

Classified as a pro-inflammatory adipokine owing to its increased expression in adipose tissue and the liver, ANGPTL4 is central to obesity and MetS onset, chiefly via lipoprotein lipase inhibition [168,169]. This, in turn, negatively regulates triglyceride metabolism and culminates in lipid accumulation and atherosclerosis onset [170,171]. Studies suggest that the deletion of ANGPTL4 in adipose tissue reduces circulating cholesterol and triglyceride concentrations, vascular inflammation, and aortic endothelial activation [172]. ANGPTL4 positively regulates IL-1β production and associated NF-Ƙβ signaling [172,173]. ANGPTL4 is chiefly triggered by factors, such as glucocorticoids and hypoxia-inducible factor-1α (HIF-1α), augmenting ANGPTL4 levels in WAT mass [173]. Additionally, ANGPTL4 is associated with serine palmitoyl transferase long chain base subunit 2 (SPTLC2) expressional upregulation, which is a key enzyme associated with ceramide production [174,175]. The activation of ceramides induces protein kinase C (PKC)-ζ dependent lipolysis, promotes pro-inflammatory mediator secretion, and leads to MetS onset [175,176,177].

##### ANGPTL8

Also known as betatrophin and lipasin, ANGPTL8 is primarily localized in the liver and adipose tissue [178]. It lacks the ANGPTL-typical fibrinogen-like domain [179]. Circulating ANGPTL8 has been reported to be augmented in systemic inflammatory response syndrome (SIRS), T2D, atherosclerosis, and NAFLD [180,181]. ANGPTL8, in combination with leukocyte immunoglobulin-like receptor B (LILRB)-2, acts as a pro-inflammatory inducer of hepatic stellate cells [182]. This, in turn, augments ERK signaling and increases genes associated with liver fibrosis [182]. ANGPTL8/LILRB2 binding on macrophages enhances the conversion of hepatic macrophages to the pro-inflammatory M1 subtype, which is primarily driven by p38/Akt/p65 phosphorylation [183]. This promotes lipid accumulation and enhances progression from simple hepatic steatosis to steatohepatitis [179,183].

#### 2.1.6. IL-6

Interleukin (IL)-6 is derived from T-cells, B-cells, and keratinocytes and is central to the regulation of a broad spectrum of biological functions [184,185]. It is a 21–28 kDa protein (exact molecular weight depends on the extent of post-translational modifications) consisting of 212 amino acids and a signal peptide of 27 amino acids [186]. Studies suggest that adipose tissue contributes 15–35% of basal circulating IL-6 [187]. IL-6 primarily functions via the activation of two signaling cascades, the JAK/STAT pathway and the MAPK pathway [188]. IL-6 binds to the IL-6 receptor (IL-6R) and leads to IL-6/IL-6R/gp130 complex formation [189]. The cytoplasmic domain of gp130 does not possess kinase activity, and the oligomerized receptor leads to the activation of tyrosine kinases of the JAK family of proteins [189,190]. This phosphorylates tyrosine residues of gp130 and opens binding sites for STAT transcription factors and Src-homology 2 (SH2) domain-containing tyrosine phosphatase 2 [191,192]. This, in turn, activates MAPK signaling [193]. The existing paradigm of studies suggests that elevated plasma IL-6 levels are associated with obesity, insulin resistance, and MetS [194,195]. In hepatocytes, a rise in IL-6 bears a close association with the onset of insulin resistance, typically via the downregulation of IRS-1 tyrosine phosphorylation and SOCS-3 expressional upsurge [196]. Adipose tissue-specific IL-6 expression has been observed to induce an inhibitory impact on insulin signaling, accompanied by the downregulation of IRS-1 and GLUT4 gene functions [197,198].

The summary of the deleterious mechanisms of some of the prominent pro-inflammatory adipokines are summarized in Table 1.

**Table 1:** Summary of the deleterious mechanisms of some of the prominent pro-inflammatory adipokines.

Adipokine	Pro-Inflammatory Mechanisms	References
TNF-α	Adipogenic effects are majorly mediated via TNFR1 binding; activates ERK-1/2, p38 MAPK, and c-JNK via MADD; activates NF-ƘB pro-inflammatory signaling and suppresses PPAR-γ (via serine phosphorylation) in adipocytes	[52,55,59]
Leptin	Promotes inflammatory cytokine upsurge in macrophages; triggers inflammatory signaling via JAK-STAT3/MAPK/PI3K signaling	[87,91,93]
Visfatin	Upregulates VEGF, FGF-2, matrix metalloproteinase, and MCP-1 production; central to NLRP3 inflammasome activation; drives arterial inflammation and endothelial damage and leads to obesity	[111,119,123]
Resistin	Downregulates AMPK signaling in the liver and skeletal muscle and interferes with insulin signaling; binds to TLR4 in the hypothalamus and mediates NF-ƘB signaling activation	[143,146]
ANGPTL2	Binds to α5β1, activates Rac1, degrades IƘB, and promotes NF-ƘB signaling activation; accelerates ICAM-1 and P-selectin expression and mediates atherosclerotic plaque formation	[164,165]
ANGPTL4	Inhibits lipoprotein lipase and culminates in lipid accumulation and atherosclerosis; positively regulates IL-1β production and NF-ƘB signaling; upregulates SPTLC2-induced ceramide production	[168,172,174]
ANGPTL8	Combines with LILRB-2 on macrophages and enhances the conversion of hepatic macrophages to M1 subtype; primarily driven by p38/Akt/p65 phosphorylation; promotes lipid accumulation and enhances progression from simple hepatic steatosis to steatohepatitis	[179,181,182]
IL-6	Activates JAK/STAT and MAPK pathways; induces insulin resistance via the downregulation of IRS-1 tyrosine phosphorylation and SOCS-3 expressional upsurge	[188,196]

### 2.2. Anti-Inflammatory Adipokines

#### 2.2.1. Adiponectin

Adiponectin is a 30 kDa adipocyte-specific adipokine that exhibits a collagen domain and a globular domain bearing homologous sequences to complement factor C1q [199,200]. As an anti-inflammatory adipokine, adipokine levels have been reported across studies to be negatively associated with plasma concentrations of pro-inflammatory CRP and IL-6 [25,26]. Adiponectin downregulates lipopolysaccharide (LPS)-induced TNF-α expression, vascular inflammation, metalloproteinase (MMP)-12 expression, and class A scavenger receptor (SR)-A in human monocyte-derived macrophages via NF-ƘB signaling inhibition [27,201,202]. This also attenuates macrophage foam cell formation [28]. Adiponectin has also been reported to reduce infiltrating CD4^+^ T lymphocyte population into atherosclerotic lesions [29]. This is driven by the downregulation of T lymphocyte chemoattractants like IF-inducing protein, IF-inducible T-cell α chemoattractant (I-TAC), and CXCl11 [23,29]. From a cardioprotection perspective, adiponectin decreases oxidative and nitrative stress and prevents inflammatory signaling post-ischemia/reperfusion injury [203]. Adiponectin is central to macrophage phenotypic switching from activated M1 phenotypes in obese and MetS subjects to anti-inflammatory M2 [204,205]. This is driven primarily via PPAR-γ-dependent signaling and holds the key to guiding macrophage ability to remove early apoptotic bodies through the calreticulin/CD91-regulating mechanism [206,207].

A large cohort of clinical studies highlights lower adiponectin among osteoporosis patients [208]. In chronic kidney disease (CKD) patients, adiponectin has been reported to be inversely correlated with cortical thickness and trabecular volumetric BMD [209]. In fact, a large body of clinical evidence shows low adiponectin levels to exhibit a strong correlation with cardiovascular complications, atherosclerosis, and MetS onset [210,211]. Low adiponectin clinically correlates with peripheral arterial stiffness in hypertension patients and compromised glycemic control in pregnancy [212,213].

#### 2.2.2. C1q/TNF-Related Protein (CTRP) Family

Primarily recognized as adiponectin structural paralogs, the CTRP family possesses a signal sequence, a C1q globular domain, and a collagen domain [214].

##### CTRP3

CTRP3, also known as cartductin or cartonectin, is primarily derived from adipocytes and mesenteric adipose tissue [215,216]. CTRP3 exhibits two alternatively spliced isoforms, CTRP3A and CTRP3B, and they differ in glycosylation and overall length [217]. It is chiefly responsible for attenuating macrophage migration inhibitory factor (MIF), MCP-1 and C-C motif chemokine ligand 4 (CCL4), TLR stimulation, and downregulating LPS-induced pro-inflammatory signaling [215,218]. Downregulated CTRP3 has been correlated with increased CCL2 and reduced adiponectin in pre-adipocytes of MetS subjects [215]. CTRP3 also improves cardiac function post-ischemia via augmenting revascularization and apoptotic reduction in an ischemic heart [219].

##### CTRP6

CTRP6, predominantly expressed in adipose tissue, circulates in the blood as heteromeric, oligomeric, or homotrimer [220,221]. CTRP6 augments anti-inflammatory IL-10 expression in monocyte-derived macrophages via p42/44 MAPK signaling activation [221]. Studies also suggest that CTRP6 activates AMPK and promotes fatty acid oxidation, regulating metabolism and inflammatory status in obese conditions [222].

##### CTRP9

CTRP9, another adipose tissue-specific adipokine, forms a heterotrimer with adiponectin and shares an AdipoR1 receptor with adiponectin [223,224]. Studies suggest that CTRP9 augments endothelial vasorelaxation in aortic rings [225]. This is driven primarily via AdipoR1/AMPK/endothelial nitric oxide synthase (eNOS) signaling [23,226]. It prevents vascular smooth muscle proliferation via protein kinase A-associated signaling [226]. CTRP9 also activates AMPK and Akt phosphorylation and increases insulin-mediated glucose uptake [223]. This attenuates the onset of insulin resistance, hepatic steatosis, and other metabolic dysfunctions [227].

##### CTRP12

Also known as adipolin, CTRP12 is a recently identified adipose tissue-derived adipokine that betters insulin sensitivity and glucose tolerance under obese conditions [228,229]. Molecular investigations reveal that CTRP12 is transcriptionally modulated by Krüppel-like factor (KLF) [230,231]. KLF3 negatively binds with the CTRP12 promoter and downregulates CTRP12 activity, outlining KLF3 as a potentially druggable target for manipulating CTRP12 transcription [232]. On the other hand, KLF-15 positively regulates CTRP12 adipocyte activity, reversing TNF-α-induced JNK dependence in obesity [233]. CTRP12 attenuates pro-inflammatory macrophage infiltration and an upsurge in the expression of TNF-α, IL-1β, and MCP-1 [229]. This is mediated primarily via Akt signaling and culminates in the downregulation of gluconeogenesis and enhances glucose uptake [23,229].

#### 2.2.3. Omentin-1

Omentin-1, a galactofuranose-binding lectin, is primarily expressed in visceral adipose tissue [234]. Studies reveal that omentin-1 is downregulated in patients exhibiting glucose intolerance, T2D, obesity, and/or in women with polycystic ovary syndrome [235,236,237]. Omentin-1 has been reported across studies to be negatively associated with metabolic dysfunction phenotypes such as dyslipidemia, hypertension, and glucose intolerance [238]. Studies suggest that omentin-1 promotes glucose uptake in adipocytes [239]. From a cardioprotective perspective, omentin-1 prevents arterial stiffness and a rise in carotid intima-media thickness [240]. This is further substantiated by reports of decreased omentin-1 in CAD [241]. Omentin-1 enhances endothelial cellular differentiation and vasodilation and reduces endothelial cell apoptosis in an AMPK/eNOS-dependent manner [242,243,244]. Omentin-1 recovers blood flow and capillary density in ischemic conditions via eNOS activation [245,246,247]. On the other hand, omentin-1 attenuates pro-inflammatory JNK signaling and avoids THP-1 monocyte adhesion to endothelial cells in response to TNF-α stimulation [248]. Omentin-1-mediated ERK/NF-ƘB signaling downregulation reduces ICAM-1 expression and attenuates p38/JNK-mediated VCAM-1 expression in vascular smooth muscle cells [249]. This avoids vascular inflammation and accounts for the cardioprotective functions of omentin-1 [250,251].

Clinical studies suggest omentin-1 as a potential biomarker for irritable bowel syndrome (IBS) patients. Beyond IBS, clinical cohort studies suggest synovial fluid-specific downregulated omentin-1 as a hallmark of rheumatoid arthritis and osteoarthritis onset [252]. In vivo studies suggest that omentin-1 supplementation betters intestinal inflammation in colitis mice, osteoporosis, and atherosclerosis [253]. Clinical cohort studies suggest that omentin-1 levels are alarmingly raised in sepsis patients [254]. Patients experiencing septic shock also exhibit higher omentin-1 and lower omentin-1 kinetics, suggesting that omentin-1 is a clinically relevant biomarker of sepsis onset and progression [254].

#### 2.2.4. Secreted Frizzled-Related Proteins (SFRPs)

Constituting a class of Wnt antagonists, SFRPs bind directly with Wnt ligands in extracellular space and intervene in both canonical and non-canonical Wnt pathways [255].

##### SFRP2

SFRP2 is an anti-inflammatory adipokine that resides in the cell matrix as well as the cytoplasm [256]. It is a 34 kDa adipokine expressed largely in adipose tissue and modulates cellular differentiation, proliferation, myocardial fibrosis, hypertrophy, and angiogenesis [257,258]. As a cardioprotective adipokine, SFRP2 attenuates cardiomyocyte apoptosis and oxidative stress and reduces ventricular fibrosis and associated myocardial infarction [259,260]. Cardioprotective activity of SFRP2 is mediated typically via Wnt/β-catenin pathway inhibition and promoting calcineurin/TFEB-driven autophagy in diabetic mice model studies [261,262]. SFRP2 also attenuates mitochondrial dysfunction in diabetic hearts and augments FUNDC1, a mitochondrial membrane protein, which binds with LC3II and activates mitophagy-induced cardiac function improvement [262,263].

##### SFRP5

SFRP5 is an anti-inflammatory adipokine that regulates metabolic dysfunction conditions [264]. Over the years, a broad range of studies suggest that SFRP5 is central to downregulating MetS conditions, such as obesity and insulin resistance, restoring endothelial nitric oxide levels in T2D [265,266,267,268]. SFRP5 exhibits anti-inflammatory activity primarily driven via Wnt5a-c-JNK (Jun N-terminal kinase) signaling inhibition [269]. Multicenter cohort studies suggest that circulating SFRP5 is lowered and correlates with worsened prognosis in heart failure and T2D patients [270]. Upregulated SFRP5 attenuates cardiac inflammation and cardiomyocyte apoptosis [271]. SFRP5 is a crucial predictor of heart failure prognosis, and lowered SFRP5 levels are corrected with vascular calcification and coronary atherosclerosis [272,273].

#### 2.2.5. Myeloid-Derived Growth Factor (MYDGF)

MYDGF, derived from chromosome 19 ORF 10, is a cardiomyocyte-protective angiogenic protein [274]. It augments the phosphorylation of BAD (at S136), Akt (at T308 and S473), and BAX (at S184) [274,275]. This downregulates cytosolic cytochrome C, cleaved caspase-9, and effector caspase-3 and caspase-7 [275]. MYDGF promotes MAPK1/MAPK3/STAT3 phosphorylation-driven endothelial cell proliferation and upregulates c-Myc/FoxM1 signaling-mediated cardiomyocyte proliferation post-heart failure [276,277,278]. MYDGF has also been reported to prevent cardiac microvascular endothelial cell apoptosis, post-ischemia/reperfusion (IR) [279]. MYDGF-ablated mice exhibit enlarged infarct size, augmented cardiomyocyte apoptosis, downregulated endothelial cell proliferation, and angiogenic responses [274]. The overexpression of MYDGF in bone marrow attenuates leukocyte influx into the aorta and prevents atherosclerosis onset via the downregulation of PKCδ/MAP4K4/NF-κB signaling [280]. MYDGF inhibits MAP4K4 phosphorylation and decreases FOXO3a signaling and LDL transcytosis, rendering protection against atherosclerosis onset [281]. In T2D, MYDGF enhances intestinal GLP-1 secretion via MAPK/MEK/ERK and PKA/GSK-3β/β-catenin signaling activation and improves insulin resistance, lipid metabolism, and glucose tolerance levels [282]. In addition, MYDGF attenuates renal oxidative stress and downregulates renal tubular cell apoptosis, inflammation, and podocyte injury via RUNX2/p27/cyclin A/CDK2 signaling activation [283,284].

Cumulatively, the metabolically protective functions of anti-inflammatory adipokines are summarized in Table 2.

## 3. Targeting Adipokines in MetS Treatment

Clinical manifestations of MetS, such as obesity, hypertension, and dyslipidemia, among others, exhibit deleterious impacts on cardiovascular homeostasis [285,286]. Adipokines, secreted primarily from adipose tissue, exhibit a substantial role in regulating metabolic and cardiovascular functions [287]. Therefore, targeting one or more of these bioactive peptides promises to design an effective therapeutic regimen against MetS conditions (Table 2).

### 3.1. Atherosclerosis and Dyslipidemia

MetS-characterized obesity is largely associated with atherosclerosis onset and is predisposed to CAD and cardiovascular disease-associated mortality [288,289,290]. Adipocyte hypertrophy without hyperplasia accounts for multiple dysfunctions such as lipid overload, adipocyte necrosis, and inflammation and culminates in atherogenic dyslipidemia onset, which is specifically characterized by triglyceride upsurge and lowered high-density lipoprotein (HDL) levels [291,292,293]. A vast majority of adipokines have been reported across studies to initiate the onset of atherosclerosis symptoms such as decreased fibrinolysis (Plasminogen Activator Inhibitor (PAI)-1), enhanced inflammation (TNF-α, IL-6), and significant insulin resistance (resistin) [294,295,296,297,298]. On the other hand, anti-inflammatory adipokine adiponectin is well recognized for its anti-atherogenic and anti-inflammatory regulatory impact [299,300]. Multiple studies over the years also suggest that dyslipidemia onset and progression are governed largely by circulating leptin and TNF-α adipokines [301]. Both leptin and TNF-α enhance the transmigration potential of low-density lipoprotein (LDL), oxidize LDL, and associate with a rise in MCP-1, VCAM-1, and ICAM-1 in endothelium [302]. Leptin-associated MCP-1 upsurge augments atherosclerotic plaque rupture and thrombus formation, culminates in coronary artery occlusion and, reduces blood supply to the heart [303]. Thus, adipokine-directed drug development assumes importance in atherosclerosis treatment.

Statins, the most widely accepted and utilized class of drugs against atherosclerosis and dyslipidemia, downregulate IL-6 expression and release, adipocyte differentiation via PPAR-γ/422Ap inhibition, C/EBPa, sterol regulatory element-binding protein (SREBP)-1, serum TNF-α, and leptin levels in adipocytes [304,305,306,307,308,309,310]. In fact, statins have also been reported to augment circulatory adiponectin concentration [311,312,313]. Fibrates, well identified as PPAR-γ activators, downregulate TNF-α mRNA and TNF-α serum concentrations in hypercholesterolemic subjects [314,315,316]. Proprotein convertase subtilisin/kexin type 9 (PCSK9) inhibitors attenuate PCSK9 from binding with LDLR [317]. This promotes a high absorption of oxidized LDLs from the bloodstream and reduces plasma LDL levels [318]. Fibrates also inhibit adipocyte-specific leptin secretion and significantly augment fatty acid oxidation and the serum concentration of anti-inflammatory adiponectin and reduce visceral adipose mass and the upregulation of uncoupling protein (UCP)-1, Positive Regulatory Domain zinc finger region protein 16 (PRDM16), peroxisome proliferator-activated receptor-γ coactivator (PGC)-1α, nuclear respiratory factor 1, and mitochondrial transcription factor A [319,320,321,322].

### 3.2. Obesity and Type 2 Diabetes (T2D)

As an increasingly serious risk factor predisposed to a broad variety of dysfunctions, obesity is a highly complicated disease condition that promotes a chronic inflammatory state and enhances insulin resistance, MetS, and the onset of T2D [323,324]. WAT expansion in obesity is characterized by ectopic fat deposition, extracellular matrix (ECM) and vascular alteration, sustained inflammation and fibrosis, damaged mitochondria, and disturbed adipokine secretion [325,326,327]. Circulating adipokines, chiefly leptin and TNF-α, are reported across studies to bear a direct correlation with dysregulated body fat deposition [328,329]. Therefore, targeting adipokines against obesity and associated T2D promises to greatly improve therapy.

Dietary and lifestyle modifications constitute the first line of therapy against obesity and correlated T2D [330]. Lifestyle interventions have been reported to reduce adiposity, mitigate T2D, augment mitochondrial biogenesis, and improve the inflammatory status of WAT [331,332]. Routine courses of aerobic and resistance exercise augment lipolysis via AMPK phosphorylation and SREBP-1c decrease [333,334]. This downregulates hepatic triglyceride synthesis, suppresses Acetyl Co-A carboxylase, and augments fatty acid oxidation and the reduction in fat mass and associated inflammatory adipokine levels [335,336]. Regular exercise promotes peripheral glucose uptake via GLUT4 translocation to skeletal muscle cell membranes [337]. This accelerates AMPK activity and, consequently, promotes insulin sensitivity [338]. Studies also suggest that chronic exercise decreases pro-inflammatory TNF-α, leptin, and MCP-1 levels [339,340]. Anti-obesity and associated T2D pharmacotherapy are currently constituted by glucagon-like peptide-1 receptor (GLP1R) agonists, biguanides (primarily metformin), thiazolidinediones, sulfonylureas, α- glucosidase inhibitors, lipase inhibitors, 5-hydroxytryptamine receptor agonists, and noradrenaline agonist/carbonic anhydrase inhibitor combination therapy, among others [341,342,343,344,345,346,347]. GLP-1R agonists typically include liraglutide and semaglutide and have been reported of late to reduce VAT adipose tissue mass [348]. GLP1R agonists also enhance adiponectin secretion and downregulate leptin levels, improving anti-inflammatory and insulin sensitivity effects [349]. Biguanides, like metformin, augment adiponectin and lower pro-inflammatory leptin and resistin and activate AMPK signaling that promotes energy metabolism and free fatty acid oxidation [350]. This downregulates ectopic fat accumulation, which enhances insulin sensitivity and avoids MetS onset [351]. To target adipokines in obesity and correlated T2D, the majority class of drugs are currently in developmental and/or clinical trial stages. These chiefly include leptin analogs, adiponectin and adiponectin receptor agonists, fibroblast growth factor (FGF)-21 analogs, IL-1R antagonists, anti-IL-1β antibodies, and palmitic acid hydroxy stearic acid (PAHSA) analogs [352,353,354,355,356]. These candidates propose to potentially lower mTOR activation, improve pancreatic β-cell function, promote gut GLP1 secretion via GPR120, and improve ceramidase activity through AdipoR1 or AdipoR2 stimulation [357,358,359].

### 3.3. Hypertension

Visceral fat accumulation constitutes one of the most prominent risk factors that is predisposed to hypertension onset [360,361,362]. Resistant hypertension is chiefly characterized by an obesity-associated rise in blood pressure despite the continued administration of multiple classes of anti-hypertensive drugs [363,364]. This has been chiefly reported to be mediated via increased aldosterone and pro-inflammatory adipokine levels [365,366]. Clinical trials of late suggest that obese subjects exhibit upregulated leptin, resistin, partial leptin resistance, and lowered adiponectin levels that cumulatively account for hypertension onset and progression [367,368,369,370]. Studies suggest proximity between resistin levels and renin–angiotensin–aldosterone system (RAAS) activity, and subjects with hypoaldosteronism are associated with increased resistin, leptin, body mass index (BMI), and deleterious modifications of cardiac morphology [371,372]. Alongside increased resistin and leptin, lowered plasma adiponectin levels are correlated with MetS cardiovascular outcomes [373]. Raised aldosterone levels downregulate adiponectin expression in adipocytes and outline RAAS/adiponectin interaction in MetS condition [374].

Existing therapeutic regimens against MetS-induced hypertension majorly target and intervene with RAAS activity [375]. Such classes of drugs primarily include angiotensin-converting enzyme (ACE) inhibitors and angiotensin receptor blockers and have been reported to better the adipokine secretion profile in hypertensive subjects [376,377]. In fact, studies suggest that mineralocorticoid receptor (MR) antagonism enhances adiponectin secretion and downregulates pro-inflammatory adipokine and inflammatory mediator(s) secretion [378]. Hyperleptinemia and associated leptin resistance, one of the precursors of MetS-induced hypertension, is associated with dysregulated sympathetic hyperactivation [377]. This suggests that the leptin receptor is a strong potential drug target for alleviating hypertension.

### 3.4. Renal Dysfunction

MetS-associated hyperlipemia and CAD cumulatively activate a complex signaling network and account for kidney damage [379]. An adipocyte-specific rise in oxidative stress drives an increase in pro-inflammatory leptin levels and hyperleptinemia, TNF-α, IL-6, IL-12, and vascular smooth muscle cell calcification [380,381]. Together, they contribute to a rise in glomerular TGF-β1 expression, endothelial cell proliferation, and collagen type IV mRNA generation and lead to focal glomerulosclerosis, proteinuria, and mesangial glucose uptake [382,383].

Although no clinically approved adipokine-directed drugs currently exist for treating renal dysfunction, ongoing developmental studies raise significant hope. MetS has been typically associated with lowered plasma adiponectin levels and increased senescent cell load, renal fibrosis, and functional impairment [384,385]. The administration of senolytics, such as Quercetin, results in a better adiponectin secretory profile, attenuates renal fibrosis, augments renal cortical oxygenation, and reduces plasma creatinine levels [386,387]. Pharmacological targeting of the renin–angiotensin system (RAS) driven by an angiotensin receptor blockade exhibits renoprotection by lowering visceral fat and WAT-specific leptin secretion [388,389]. Combining spironolactone with ACE inhibitors promises to decrease albuminuria and obesity-associated renal dysfunction [390]. Studies also suggest that sodium/glucose co-transporter (SGLT)-2 inhibitors have potential to better MetS-induced kidney injury [391]. They attenuate glomerular hyperfiltration and impede renal injury progression [392]. This can potentially improve glomerular and tubular hemodynamics and culminate in renoprotection [393,394]. However, further mechanistic understanding of SGLT2 inhibition needs to be undertaken to potentially define their interaction (if any) with the adipokine profile and better explain associated renoprotection.

### 3.5. Osteoporosis

Osteoporosis, a global health concern for the elderly population, is largely characterized by reduced bone density and dysregulated bone structural integrity [395,396]. The existing paradigm of studies suggests that adipokines influence bone mineral density, cortical thickness, and fracture [396]. Pro-inflammatory adipokines, like leptin and resistin, bear a strong association with poor bone strength [397]. Increased circulating resistin increases osteoclastic activity and promotes bone remodeling and resorption via NF-Ƙβ signaling activation [398,399]. Clinical studies suggest that resistin levels, along with IL-1 and IL-6, are augmented in post-menopausal women and serve as predisposing factors for osteoporosis onset [400]. Leptin mRNA levels have been reported to be augmented in the cartilage and synovial fluid of osteoporosis and osteoarthritis patients [401]. Leptin activates c-Myc and PKA/ATF4/RANKL signaling that downregulates osteoblast proliferation and promotes bone resorption of osteoclasts [402]. This culminates in chondrocyte hypertrophy and excessive ossification [403]. Similarly, inflammatory adipokine visfatin has been reported to promote bone catabolism and negatively influence osteoblastic glucose uptake and collagen synthesis [404].

A clear understanding of the link between adipokines and bone health promises to improve therapeutic regimens against osteoporosis-associated fracture risk and bone degeneration [405]. Pre-clinical studies propose that the ablation of chemerin or its receptor chemokine-like receptor 1 (CMKLR1) attenuates adipocyte differentiation, mesenchymal stem cell proliferation, and augmented osteoblast gene expression and mineralization with an osteoblastogenic stimulus [406]. Such studies also highlight that chemerin/CMKLR1 modulates alterations in adipogenic and osteoblastogenic transcription factors that interfere with Wnt signaling [407]. Moreover, pre-clinical studies have shown that anti-inflammatory adipokine omentin-1 attenuates estrogen deficiency-induced bone loss by RANK/OPG downregulation and augments osteoblast differentiation via TGF-β/Smad signaling. This, in turn, also increases the expression of transcription factors collagen1, osteopontin, osteocalcin, and osterix [408,409]. Currently, ongoing studies involving human subjects shall better explain the therapeutic potential of adipokines in osteoporosis management.

### 3.6. Non-Alcoholic Fatty Liver Disease (NAFLD)

NAFLD is chiefly characterized by upregulated hepatic fat mass (steatosis) in the absence of alcohol intake [408,409]. Genetic alterations, gut microbiome dysregulation, and metabolic abnormalities are some of the key predisposing factors of NAFLD onset and culminate in hypertriglyceridemia, reduced HDL, hypertension, and/or high fasting glucose levels [410]. Adipose tissue and the liver exhibit a critically coordinated role in glucose and lipid metabolism and immunological and energy homeostasis in the body [411,412]. In obese conditions, ectopic lipid accumulation starts in the liver since WAT reaches its limit of lipid storage [413].

A large body of studies suggest the potential role of adipokines in NAFLD onset and progression [412]. Pre-clinical studies show that high plasma leptin is strongly correlated with hepatic inflammatory and fibrogenetic processes that culminate in NAFLD onset [414]. NAFLD murine model studies also reveal high plasma resistin that alters mitochondrial physiology and upregulates pro-inflammatory mediators that aggravate NAFLD-associated steatosis by AMPK/PGC-1α signaling [415]. Similarly, visfatin exacerbates NAFLD-induced hepatic steatosis, inflammatory cytokine levels, and cellular infiltration mediated by increased ER stress [416]. On the contrary, anti-inflammatory adipokine adiponectin attenuates NAFLD-induced hepatic inflammation, steatosis, and fibrosis [417]. Adiponectin reduces NAFLD-associated pro-inflammatory signaling via TLR4 modulation [417]. Pharmacological studies aimed at developing NAFLD therapy identified biguanides, chiefly metformin, in preventing hepatic triglyceride accumulation and NAFLD onset [418]. Randomized controlled trials with thiazolidinediones suggest that thiazolidinediones (chiefly Rosiglitazone and Pioglitazone) downregulate hepatic steatosis and lobular inflammation in non-diabetic NAFLD subjects [419]. Thiazolidinediones augment adiponectin secretion that controls the visceral/subcutaneous fat mass ratio. Additionally, thiazolidinediones also reduce leptin levels in NAFLD subjects [420]. However, further pathophysiological studies with enhanced clinical insights shall enable a better comprehension of adipokine therapeutic potential for NAFLD treatment.

Cumulatively, the summary of the existing adipokine-targeted therapeutic regimens for MetS treatment are summarized in Table 3.

**Table 3:** Brief summary of the existing adipokine-targeted therapeutic regimens for MetS treatment.

MetS Condition	Adipokine-Targeted Therapeutic Intervention	References
Atherosclerosis and Dyslipidemia	(a) Statins—downregulate IL-6, adipocyte differentiation, adipocyte leptin, and TNF-α (b) Fibrates—downregulate visceral adipose mass, TNF-α serum concentration, and mRNA and promote fatty acid oxidation, serum adiponectin levels, and UCP-1 (c) PCSK9 inhibitors—attenuate PCSK9 from binding with LDLR, promote a high absorption of oxidized LDLs from the bloodstream, and reduce plasma LDL levels	[305,308,314,316,318]
Obesity and T2D	(a) Lifestyle modifications—reduce adiposity, promote mitochondrial biogenesis, and improve WAT inflammatory status (b) Adiponectin receptor agonists—improve pancreatic β-cell function (c) Palmitic acid hydroxy stearic acid (PAHSA) analogs—improve ceramidase activity via AdipoR1 or AdipoR2 stimulation (d) GLP1R agonists—enhance adiponectin secretion and downregulate leptin levels; improve anti-inflammatory and insulin sensitivity effects (e) Biguanides—augment adiponectin and lower pro-inflammatory leptin and resistin; activate AMPK signaling that promotes energy metabolism and free fatty acid oxidation	[347,348,350,356,357,359]
Hypertension	(a) ACE inhibitors—improve adipokine profile (b) MR antagonists—promote adiponectin secretion; downregulate pro-inflammatory adipokine release	[376,377,378]
Renal Dysfunction	(a) Senolytics—improve adiponectin profile, attenuate renal fibrosis, and augment renal cortical oxygenation (b) RAS inhibitors—lower visceral fat and WAT leptin secretion (c) SGLT2 inhibitors—prevent glomerular hyperfiltration and hinder renal damage progression	[386,388,392]
Osteoporosis	(a) Omentin-1 supplementation—attenuate estrogen deficiency-induced bone loss by RANK/OPG downregulation; augment osteoblast differentiation via TGF-β/Smad signaling (b) Chemerin inhibition—attenuate adipocyte differentiation, mesenchymal stem cell proliferation, and augmented osteoblast gene expression and mineralization	[406,408]
Non-Alcoholic Fatty Liver Disease (NAFLD)	(a) Biguanides—prevent hepatic triglyceride accumulation (b) Thiazolidinediones—downregulate hepatic steatosis and lobular inflammation; promote adiponectin secretion that controls the visceral/subcutaneous fat mass ratio	[418,420]

## 4. Current Limitations and Future Landscape

Obesity and associated metabolic disorders are well-established precursors to a broad variety of neurodegenerative, respiratory, and cardiovascular complications [1,421,422]. Aspiring to improve MetS therapy, the role of adipokines assumes great significance in better explaining the mechanisms via which MetS initiates and amplifies associated dysfunction [423,424]. The manipulation of the adipokine secretion profile promises great potential for ameliorating MetS [299,425]. However, adipokine-directed therapy currently faces significant challenges. In developing novel drug candidates to better adiponectin secretion profiles, adiponectin protein stability, restricted circulatory half-life, and susceptibility to gastrointestinal enzymatic degradation constitute major concerns [426,427,428]. A lack of tissue specificity and diverse physiological adipokine activities are predisposed to unprecedented adverse effects of adipokine-directed therapy [429,430]. The determination of the optimal drug dose and the understanding of the long-term physiological impact of adipokine-targeted therapy remain largely unclear to date [431,432]. The genetic polymorphisms of the ADPOQ gene and associated inter-individual differences, the potential for drug–drug interactions, and comorbid health status complicate our understanding even further [433,434,435,436].

Addressing the above-mentioned concerns assumes importance and constitutes the future landscape of adipokine-directed therapy development. Liposome-mediated drug delivery can be focused on augmenting circulatory half-life and improving the therapeutic profile of the designed drug [437]. Existing genetic polymorphisms point towards developing personalized therapy, relying on individual adiponectin secretion profiles [438]. Extensive pharmacokinetic and pharmacodynamic studies need to be undertaken to better understand drug–drug interactions and the impact of dysfunctional metabolism on the adipokine profile [439]. Multicentric clinical trials can potentially better explain adipokine-directed therapeutic efficacy across patient cohorts [440]. Additionally, developing adiponectin analogs promises to be a great step toward confronting existing limitations with the adiponectin profile [429].

## 5. Conclusions

The existing paradigm of studies suggests that the adipokine secretory profile is a strong mediator of MetS onset and correlated physiological damage. The signaling networks regulated by pro-inflammatory and anti-inflammatory adipokines constitute a uniquely complicated cascade for driving or hindering inflammatory response. Understanding these signaling cross-talks can potentially empower translational investigations to better comprehend adipokine-directed therapy in MetS conditions. Studies propose adipokine-targeted therapy as a promising strategy for mitigating MetS and associated complications. However, existing limitations pose major challenges for the clinical implication of such therapy. Addressing these scientific gaps and amalgamating them with advanced genomics and metabolomic investigations coupled with greater courses of translational research can potentially enable clinical implications and ensure a better understanding of precision-driven adipokine-targeted MetS therapy in the near future.

## Figures and Tables

**Table 2:** Summary of the metabolically protective functions of some of the prominent anti-inflammatory adipokines.

Adipokine	Protective Anti-Inflammatory Function	References
Adiponectin	Downregulates LPS-induced TNF-α expression, vascular inflammation, MMP-12 expression, and SRA in monocyte-derived macrophages via NF-ƘB signaling; reduces CD4^+^ T lymphocyte infiltration into atherosclerotic lesions and ischemia/reperfusion-induced oxidative stress	[25,29,202]
CTRP3	Attenuates MIF, MCP-1, CCL4, TLR upregulation, and LPS-induced pro-inflammatory signaling; promotes revascularization and apoptotic downregulation in ischemic heart	[215,218,219]
CTRP6	Upregulates IL-10 expression via p42/44 MAPK signaling stimulation; activates AMPK and fatty acid oxidation in obesity	[221,222]
CTRP9	Enhances vasorelaxation in aortic rings via AdipoR1/AMPK/eNOS signaling; attenuates protein kinase A-driven vascular smooth muscle cell proliferation	[223,226,227]
CTRP12	Improves insulin sensitivity and glucose tolerance in obesity; downregulates macrophage infiltration and rise in TNF-α, IL-1β, and MCP-1 via Akt signaling activation	[228,229]
Omentin-1	Promotes adipocyte glucose uptake, enhances endothelial differentiation, and attenuates endothelial apoptosis by AMPK/eNOS signaling activation; downregulates pro-inflammatory JNK and ERK/NF-ƘB signaling in vascular smooth muscle cells	[239,242,248]
SFRP2	Inhibits the Wnt/β-catenin pathway; promotes calcineurin/TFEB-driven autophagy in diabetic mice; attenuates mitochondrial dysfunction in diabetic hearts; augments FUNDC1/LC3II binding and mitophagy that improves cardiac function	[261,262,263]
SFRP5	Inhibits Wnt-5a/c-JNK signaling; attenuates cardiomyocyte apoptosis, cardiac inflammation, coronary atherosclerosis, and vascular calcification	[269,271,272,273]
MYDGF	Promotes MAPK1/MAPK3/STAT3 phosphorylation (endothelial cell proliferation) and c-Myc/FoxM1 signaling (cardiomyocyte proliferation) post-heart failure; prevents atherosclerosis onset via PKCδ/MAP4K4/NF-κB downregulation; enhances intestinal GLP-1 secretion via MAPK/MEK/ERK and PKA/GSK-3β/β-catenin activation and improves insulin resistance, lipid metabolism, and glucose tolerance	[276,280,282]

## Data Availability

No new data were created or analyzed in this study.
